# Carbon-bridged oligo(*p*-phenylenevinylene)s for photostable and broadly tunable, solution-processable thin film organic lasers

**DOI:** 10.1038/ncomms9458

**Published:** 2015-09-29

**Authors:** Marta Morales-Vidal, Pedro G. Boj, José M. Villalvilla, José A. Quintana, Qifan Yan, Nai-Ti Lin, Xiaozhang Zhu, Nopporn Ruangsupapichat, Juan Casado, Hayato Tsuji, Eiichi Nakamura, María A. Díaz-García

**Affiliations:** 1 Departamento Física Aplicada and Instituto Universitario de Materiales de Alicante, Universidad de Alicante, Alicante 03080, Spain; 2 Departamento Óptica, Farmacología y Anatomía; and Instituto Universitario de Materiales de Alicante, Universidad de Alicante, Alicante 03080, Spain; 3 Department of Chemistry, School of Science, The University of Tokyo, Hongo, Bunkyo-ku, Tokyo 113-0033, Japan; 4 Department of Physical Chemistry, University of Málaga, Andalucía Tech., Campus de Teatinos s/n, Málaga 29071, Spain; 5 JST-PRESTO, 4-1-8 Honcho, Kawaguchi, Saitama 332-0012, Japan

## Abstract

Thin film organic lasers represent a new generation of inexpensive, mechanically flexible devices for spectroscopy, optical communications and sensing. For this purpose, it is desired to develop highly efficient, stable, wavelength-tunable and solution-processable organic laser materials. Here we report that carbon-bridged oligo(*p*-phenylenevinylene)s serve as optimal materials combining all these properties simultaneously at the level required for applications by demonstrating amplified spontaneous emission and distributed feedback laser devices. A series of six compounds, with the repeating unit from 1 to 6, doped into polystyrene films undergo amplified spontaneous emission from 385 to 585 nm with remarkably low threshold and high net gain coefficients, as well as high photostability. The fabricated lasers show narrow linewidth (<0.13 nm) single mode emission at very low thresholds (0.7 kW cm^−2^), long operational lifetimes (>10^5^ pump pulses for oligomers with three to six repeating units) and wavelength tunability across the visible spectrum (408–591 nm).

## Abstract (web summary)

Thin film organic solid-state lasers are low-cost flexible devices which require efficient, stable, colour-tunable, solution-processable materials. Here, the authors show that oligo(p-phenylenevinylene)s simultaneously possess all such properties, as demonstrated by their use in laser devices.

## Graphical abstract

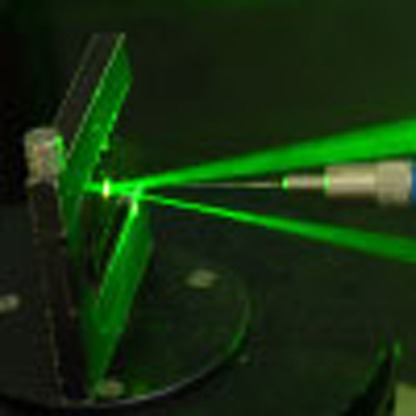

A distributed feedback (DFB) laser prepared as a thin film waveguide (in-plane light propagation) by a solution process represents an attractive thin film organic laser (TFOL) device^1^,^2^ for numerous applications^3^,^4^,^5^,^6^,^7^ for various reasons: single mode emission, low pump energy and easy integration of the resonator into other devices, as well as mechanical flexibility and potentially low production cost. The optical feedback is achieved generally by a relief grating, patterned typically by lithographic techniques, either on a substrate on which the active material is coated, or more economically on a film of the active material itself by nanoimprint lithography (NIL)^8^.

With highly promising device architectures available, the major challenge remaining is to develop active organic materials, which are required to be photostable to ensure a long operational lifetime for the device, efficient for lasing at a low threshold to operate under excitation with a weak light source and capable of emission at various wavelengths or colours (colour tuning). A variety of active organic materials have been developed, generally focusing on improving each of these parameters, but rarely on systematic synthetic design aiming at optimizing all of them simultaneously. Most of the materials thus far investigated^1^,^2^ belong to one of the two following categories: organic semiconductors—mainly polymers, such as polyphenylenevinylenes or polyfluorenes, as well as oligomers, dendrimers and small molecules, all of which were used as a neat film; and conventional aromatic dyes dispersed typically at 3–5 wt% in an inert solid matrix among which thermoplastic polymers are the most convenient for processing.

Studies on organic semiconductors have largely focused on the lowering of the laser threshold, and less frequently on the improvement of their photostability. For instance, many organic semiconductors, coated on DFB gratings, show threshold values <1 kW cm^−2^ (refs 1, 2, 9, 10, 11, 12, 13, 14, 15), which provide people with a prospect of pumping with a light-emitting diode (LED) instead of with a laser^13^,^14^,^15^. Their photoinstability under ambient conditions and the resulting short operational lifetime, however, necessitate protection of the device from molecular oxygen, except in few cases^12^. On the other hand, TFOL devices using certain aromatic dyes, particularly pyrromethenes^16^ and perylenediimides (PDIs)^17^,^18^,^19^,^20^, dispersed in polystyrene (PS) or poly(methylmethacrylate) (PMMA), show operational lifetimes as long as 10^5^ pump pulses (pp)^17^, whereas their thresholds are often high, typically 10–100 kW cm^−2^ (refs 1, 2, 16, 17, 18, 20) and 3 kW cm^−2^ at the lowest^19^. The higher photostability and threshold values of these materials are a consequence of the high dye dilution in the matrix that it is necessary to prevent aggregative excited state annihilation. Therefore, there have been evident needs to develop a new molecular design and a synthetic strategy for achieving systematic colour tuning^21^,^22^,^23^, high photostability and low threshold stimulated emission at the same time.

Here we report that carbon-bridged oligo(*p*-phenylenevinylene)s (COPVs)^24^ are new organic dyes where the above-mentioned impediments are largely eliminated, making them unique among known laser organic systems. A completely flat and robust all-carbon skeleton is available here in a homologous series of COPV*n* with a repeating unit *n* from 1 to 6, which can be excited with low photoexcitation energy^25^,^26^,^27^. A notable structural feature of these molecules is the presence of bulky aromatic side chains (that is, *p*-octylphenyl groups) on both sides of the flat π-system which sterically prevent molecular aggregation, intermolecular chemical reactions and quenching of the photoexcited states. These bulky substituent groups also endow these compounds with high solubility in a variety of organic solvents—unusual property for highly conjugated π-systems. In addition, the substituents also endow the compounds with high miscibility in an organic solid matrix, which facilitates fabrication as thin films with good optical and mechanical quality, and maximizes the dye load without aggregation. Another prominent property of COPV*n* is the high film photoluminescence (PL) efficiency, as well as exceptionally high stability of multipolarons (up to tetrapolaron) for COPV*n* with *n*≥3 (ref. 24). The conjugation and the steric protection invoke stabilization of the photoexcited state and resistance to degradation under intense illumination such as in lasing. Reported below is the high performance for laser purposes of the new π-system, represented by the data for COPV6 that shows ASE emission between 582 and 585 nm: a very low ASE threshold (*I*_th-ASE_<2 kW cm^−2^) and an exceptionally long ASE photostability half-life (*τ*_1/2_^ASE^) of *ca*. 1 × 10^6^ pp, or >24 h, under a pulsed optical pump operating at a repetition rate of 10Hz; as well as a very low DFB threshold (*I*_th-DFB_=0.7 kW cm^−2^, *ca*. 70 nJ pulse^−1^) and operational lifetime as long as *τ*_1/2_^DFB^=1 × 10^5^ pp. COPV6 thus shows simultaneously a lower DFB threshold (*I*_th-DFB_) and a longer lifetime than the hitherto known organic laser dyes.

## Results

### ASE properties of COPV*n* dispersed in PS films

**Figure 1:**
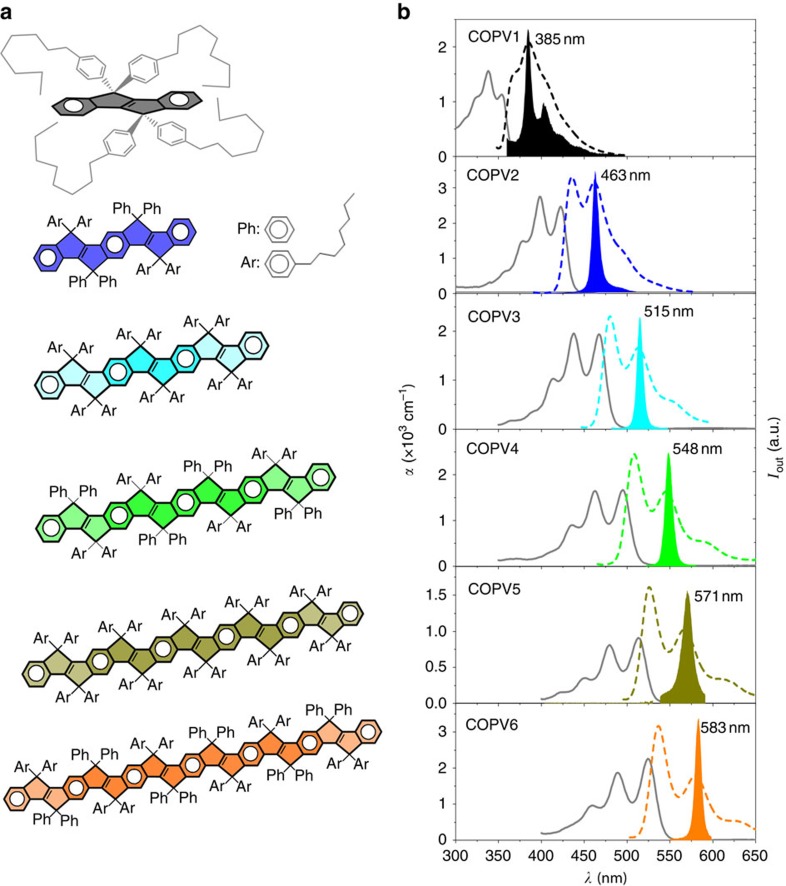
Chemical structures of COPV*n* (*n*=1 to 6) and optical properties of COPV*n* dispersed in polystyrene films. (**a**) Chemical structures of COPV1–6, from top to down, including a three-dimensional perspective for COPV1. (**b**) Absorption coefficient, *α* (solid line, left axis), photoluminescence intensity (dashed line, right axis) and amplified spontaneous emission, ASE, intensity (filled area, right axis), versus wavelength, *λ*, for films doped with COPV1–6 (doping rates 3 wt%, 3 wt%, 2 wt%, 1.7 wt%, 1 wt% and 2 wt%, respectively) deposited over fused silica. The ASE wavelength value for each compound is indicated in its corresponding figure.

Thin films (∼600-nm thick) of COPV*n*, for *n*=1–6 (chemical structure in Fig. 1a), dispersed in PS, used as passive matrix, were prepared. A toluene solution containing a COPV*n* derivative and PS was spin-coated on a 1-mm-thick transparent fused silica (FS) substrate. The COPV*n* concentration with respect to PS varied from 0.5 to 5 wt%, and even up to 15 and 20 wt% for COPV4 and COPV6, respectively.

A study of the ASE properties indicated that the emission wavelength (*λ*_ASE_) covers a wide range of the visible spectrum, that is, from 385 nm in COPV1, up to 585 nm in COPV6 (Fig. 1b and Table 1). The tunability of *λ*_ASE_ through a simple change in the number of repeating units (for example, Fig. 1a) represents an important advantage of the COPV dye system as compared to the conventional dye tuning strategy, in which entirely different chemical structures are needed to cover an equivalent range of the visible light spectrum (for example, PDIs with good laser performance cover only between 580 and 620 nm)^18^,^19^. A similar strategy of tuning the laser wavelength by increasing the number of repeating units has been widely employed for structurally flexible π-conjugated oligomers^22^,^28^ and polymers^21^, which, however, increased their structural mobility, significantly reduced their chemical stability, and hence impeded their ASE thresholds and lifetimes.

**Figure 2:**
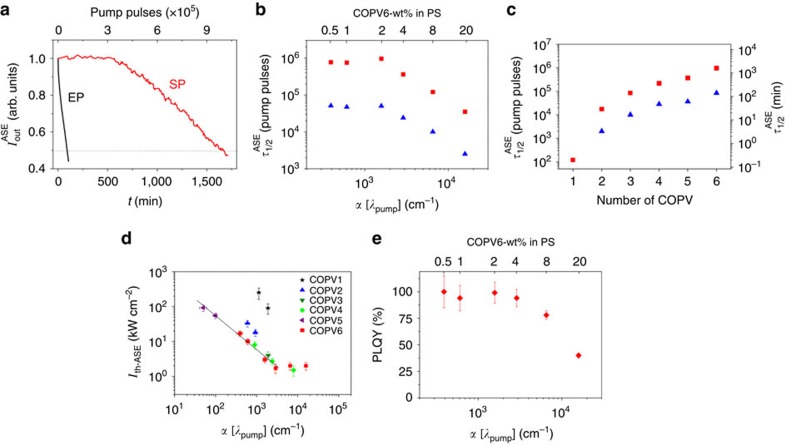
Amplified spontaneous emission (ASE) properties of COPV*n* (*n*=1 to 6) dispersed in polystyrene films. (**a**) ASE intensity, I_out_^ASE^, versus time, *t*, and versus the number of pump pulses (bottom and top axes, respectively) for a 2-wt% COPV6-doped film, excited continuously under soft pump (SP, full line, (I_pump_/I_th-ASE_)∼2) and extreme pump (EP, dashed line, I_pump_=2.5 × 10^3^ kW cm^−2^, (I_pump_/I_th-ASE_)∼10^3^) conditions. (**b**) ASE photostability half-life, *τ*_1/2_^ASE^, versus the absorption coefficient at the pump wavelength, *α*[*λ*_pump_], of films with different COPV6 concentrations (shown on the top axis) under SP (▪) and EP (▴) conditions. (**c**) *τ*_1/2_^ASE^ values for all COPV*n* (doping rates into PS are 3 wt%, 3 wt%, 2 wt%, 1.7 wt%, 1 wt% and 2 wt%, for COPV1–6 respectively) under EP (▪) and SP (▴) conditions. (**d**) ASE thresholds, I_th-ASE_, for all COPV*n* versus *α*[*λ*_pump_]. The full line is a guide to the eye to show the behaviour trend for COPV3–6. (**e**) Film photoluminescence quantum yield, PLQY, for COPV6 versus *α*[*λ*_pump_] and versus dye concentration (bottom and top axes, respectively). Errors in *α*[*λ*_pump_], I_th-ASE_, *τ*_1/2_^ASE^ and PLQY were estimated statistically as the s.d. from measurements on several nominally identical samples. Error bars for *τ*_1/2_^ASE^ not shown because they are two small (error∼10%) in the logarithmic scales used in the graphs.

The high photostability of COPV*n* under ambient conditions is a remarkable feature. A 2-wt% COPV6 film (Fig. 2a) under air at room temperature shows a *τ*_1/2_^ASE^ as large as 1 × 10^6^ pp, or >24 h, under soft pumping (SP) conditions, that is, under a pump intensity (*I*_pump_) only twice that of *I*_th-ASE_ (*ca*. 3 kW cm^−2^, or 520 nJ per pulse). This photostability is in stark contrast to that of flexible counterparts of COPVs, such as phenylenevinylene oligomers^28^ and polymers^1^,^2^, whose ASE under ambient conditions lasts for just a few minutes. Moreover, the photostability lifetimes of COPV*n* are even better than the best results reported for aromatic laser dyes doped in a polymer matrix^16^,^17^,^18^,^19^,^20^. The high photostability of COPV*n* was further attested by an experiment pumping COPV6 with an extremely intense light (Fig. 2a), denoted here as extreme pump (EP) conditions (*I*_pump_=2.5 × 10^3^ kW cm^−2^, ∼10^3^ times more intense than its threshold), where *τ*_1/2_^ASE^ decreased only by a factor of 20 (5.5 × 10^4^ pp, *ca*. 92 min).

Dye laser photostability, which is intrinsically related to intermolecular degradation pathways, depends on the concentration of the dye in the polymer matrix, that is, on the absorption coefficient of the film at a given pump wavelength, *α*[*λ*_pump_], as has been observed in PDIs dispersed in PS and PMMA^18^,^19^. The half-life *τ*_1/2_^ASE^ of COPV6, therefore, decreases when *α*[*λ*_pump_] increases (Fig. 2b), yet even at a doping rate as heavy as 20 wt% the lifetime is still long (*τ*_1/2_^ASE^=3.5 × 10^4^ pp).

COPV3–5 are likewise highly photostable (*τ*_1/2_^ASE^∼10^5^ pp under SP, Fig. 2c) in air at room temperature, while *τ*_1/2_^ASE^ decreases gradually from COPV6 to COPV3. On the other hand, the photostability decreases significantly for COPV1 and COPV2, probably because of their high photoexcited state energy. COPV1 decomposed too quickly under EP conditions to be studied for its half-life. We consider that the high photostabilities of COPV3–6 reflect the robustness of their excited states—a property probably originating from the steric protection and the effective π-conjugation, as demonstrated by Raman spectroscopy of the neutral and cationic COPVs^29^. Experiments performed under a nitrogen atmosphere for COPV1–2 and COPV6 showed that the half-life increases by two times, suggesting that the degradation process involves air oxidation.

The correlation between *α*[*λ*_pump_] and *I*_th-ASE_ provides further support for the difference between COPV1–2 and COPV3–6 (see Table 1 for physical, optical and ASE parameters; and Supplementary Fig. 1 for details on threshold determination). In Fig. 2d, we find that the data for each of the compounds show an inverse correlation between *α*[*λ*_pump_] and *I*_th-ASE_, and all data except for COPV1–2 and highly doped COPV6 (that is, with larger *α*[*λ*_pump_]) are aligned on the same slope. Taken together with the *τ*_1/2_^ASE^ data in Fig. 2c and the adverse effects of molecular oxygen for ASE (see above), we suggest that the higher *I*_th-ASE_ values and the shorter operational times of COPV1 and COPV2 are related to activation of the excited state because of photoreaction through the unprotected terminal positions, similarly to the reaction with molecular oxygen.

The PL quantum yield (PLQY) for all COPV*n* derivatives in PS films remains, as in solution^24^, extremely high (>90%) up to 5 wt% (see Table 1) and decreases for higher doping rates (see data for COPV6 in Fig. 2e) suggesting that the fluorescence quenching is because of intermolecular aggregation. In fact, the correlation found between *α*[*λ*_pump_] and *I*_th-ASE_ in Fig. 2d and between *α*[*λ*_pump_] and PLQY in Fig. 2e suggests that intermolecular excited state annihilation already appears as a limiting factor at a high doping ratio. A similar correlation has been observed with PDI-doped PS films^18^. Net gain coefficients (*g*) of 60 and 6.3 cm^−1^, at pump intensities of 43.3 and 11.5 kW cm^−2^, respectively, were determined for the 8-wt% COPV6-doped film by means of a variable stripe length study (see Supplementary Fig. 2 and Supplementary Note 1 for details). These *g* values are much superior to those obtained with other *p*-phenylenevinylene oligomers (*g*=13 cm^−1^ at *I*_pump_=55 kW cm^−2^)^28^ and PDIs dispersed in PS (*g*=8 cm^−1^ at *I*_pump_=60 kW cm^−2^)^30^ and only about twice lower than state-of-the-art organic semiconductors^12^,^31^. Overall, we conclude that COPV*n* show uniformly good and often much better performance simultaneously on *τ*_1/2_^ASE^ and *I*_th-ASE_ at their respective ASE wavelengths than previously known materials^1^,^2^,^9^,^10^,^11^,^13^,^14^,^15^,^16^,^17^,^18^,^19^,^20^,^21^,^22^,^23^.

### DFB lasers based on COPV*n*-doped PS films

**Figure 3:**
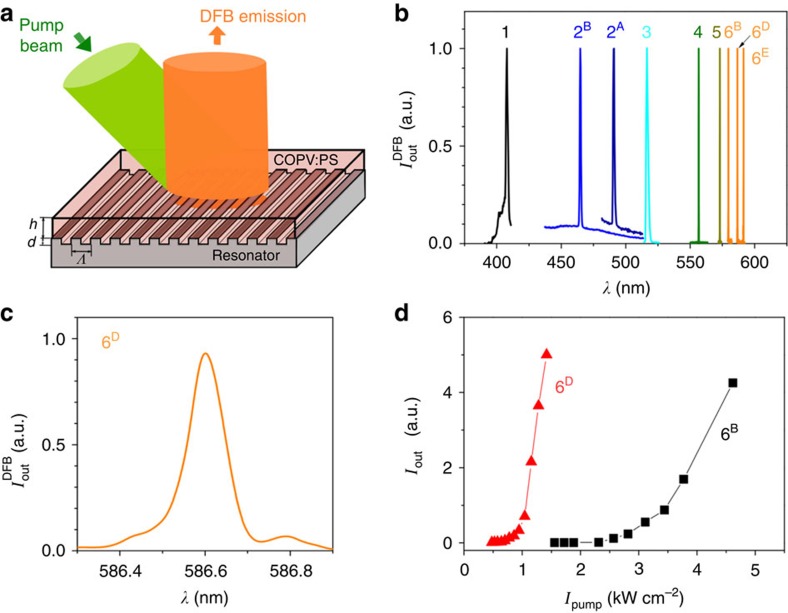
Laser properties of COPV*n* (*n*=1 to 6) distributed feedback (DFB) lasers. (**a**) Sketch of the DFB device (*Λ*, grating period; *d*, grating depth; *h*, active film thickness) and excitation/collection geometry. (**b**) Spectra of DFB lasers based on COPV*n*-doped polystyrene films (the number on the device label denotes *n*) deposited over different types of resonators. Device parameters (COPV*n* doping rate, resonator material, *Λ*, *d* and *h*) are listed in Table 2. (**c**) DFB spectrum for device 6^D^ on an expanded scale. (**d**) Output intensity versus pump intensity for devices 6^B^ (▪) and 6^D^ (▴). Full lines are guides to the eye.

The ASE performance of COPV*n* can be fully exploited for the fabrication of DFB lasers (Table 2), which simultaneously show a low threshold *I*_th-DFB_, long operational half-life *τ*_1/2_^DFB^ and wide wavelength tuning capability, superior to known organic systems. COPV*n*-doped PS was spin-coated over resonator substrates with one-dimensional gratings (device scheme in Fig. 3a). The grating periods (*Λ*) were chosen to produce lasers operating in the second order, thus emitting in a direction perpendicular to the film surface^1^,^2^. DFB gratings for COPV*n* (*n*=3–6) were engraved, prior to the COPV film deposition, by thermal-NIL and subsequent etching on FS substrates, or alternatively on transparent 1-μm-thick SiO_2_ layers grown by thermal oxidation over silicon wafers (denoted as SiO_2_ substrates). For COPV*n* (*n*=1 and 2) devices, gratings were fabricated by holographic lithography (HL) on photoresist layers deposited over FS substrates, or by HL and subsequent etching over glass. A list of relevant geometrical and performance parameters for the DFB lasers is in Table 2 and representative DFB spectra are shown in Fig. 3b. We focus now mainly on the best performing system, COPV6. The lasing wavelength *λ*_DFB_ was tuned (Table 2, devices 6^A^ to 6^E^) within a spectral range of ∼20 nm centred at the wavelength of maximum gain (*λ*_ASE_) by changing *Λ* and/or the thickness of the COPV film (*h*). Single mode emission was obtained in all cases with linewidths <0.13 nm (Fig. 3c). A detailed discussion about the spectral shape dependence on *h*, *d* and on the size of the pump beam over the sample was recently reported for PDI-doped PS DFB lasers with the same resonator type and device parameters used here for COPV3–6 (ref. 32). Devices based on 8-wt% COPV6-doped films, emitting close to *λ*_ASE_ (devices 6^C^ and 6^D^, Table 2; 6^D^ also in Fig. 3d) have shown a *I*_th-DFB_ as low as 0.7±0.1 kW cm^−2^, or 70±10 nJ per pulse, this value being the lowest among the reported DFBs based on dye-doped polymer-active materials, and very close to the requirements for LED pumping^14^,^15^. In addition, these devices at 8 wt% doping recorded operational lifetimes as long as *τ*_1/2_^DFB^=1.0 × 10^5^ pp. A COPV laser prepared with 2 wt% of COPV6 showed a longer lifetime (device 6^B^ in Table 2, *τ*_1/2_^DFB^=1.0 × 10^6^ pp) at the expenses of a slightly higher threshold value of *I*_th-DFB_=2.1±0.2 kW cm^−2^ or 210±20 nJ per pulse (Fig. 3d). The higher DFB threshold of device 6^B^ is mainly due to the lower COPV content and consequently lower absorption coefficient and higher ASE threshold as shown in Fig. 2d. It is also because the separation of *λ*_DFB_ from *λ*_ASE_ of this device is larger than that of device 6^C^ (Table 2) as this parameter is known to exert the largest influence on the threshold^20^,^32^. The slope efficiency of the 8-wt% COPV6 DFB lasers is estimated to be <2%, a value lower than those reported for some DFBs based on neat films of semiconducting polymers^11^. It is largely because of 1 order of magnitude smaller absorption coefficients of the COPV6 films due to the dilution in the polymer matrix, which in turn contributes to improve their photostability. DFB data for COPV1–5 are also shown in Table 2 and Fig. 3b, illustrating the utility of COPV*n* for a DFB laser operating over a wide visible light region (408–591 nm).

Finally, we note the thermal stability of COPV*n*. The decomposition temperature of COPV6 is 439 °C (5% weight loss, data and details in Supplementary Fig. 3), and the PL and ASE properties of a COPV-doped PS film remain unchanged after heating at 155 °C for 15 min—thermal conditions similar to those used for thermal-NIL processing. Thus COPVs far surpass, in their thermal stability, the state-of-the-art conjugated polymers that are thermally labile, and rival thermally and structurally robust PDI derivatives^33^. Hence, we expect that COPVs will allow thermal-NIL imprinting of the DFB resonators directly onto the active film, as previously demonstrated for PDI-doped PS^20^.

## Discussion

In summary, COPV*n* are optimal hybrids of conjugated polymers and small-molecule aromatic dyes for lasing action because they amalgamate the best properties of each, made possible by the all-carbon, flat, large and rigid molecular framework protected by bulky aryl substituents. Their conjugation length can be tuned precisely by way of scalable chemical synthesis. Unlike aromatic dyes, COPVs allow to systematically tune the laser wavelength output over a wide range of the visible spectrum. Their planar π-conjugated core is ideal for maximal PL and consequently low ASE threshold. The *p*-octylphenyl substituents, which can be changed to a number of other groups by synthetic design^25^, provide solubility in common organic solvents, and hence processability as thin films by solution-based methods. They also impart protection of the π-system and further minimize chemical degradation and self-aggregation, in favour of very long laser operational lifetimes. The DFB lasers prepared in this work already satisfy the various requirements needed for applications—wavelength tunability, operational lifetime, threshold, processability and fabrication cost. Importantly, there is still plenty of room for improvement of the various laser parameters and for further technological developments; for example, the use of substructured DFB gratings^34^, optimization of the excitation geometry^14^,^35^, the excitation wavelength or the polymer matrix^36^, might allow improving the threshold and operational lifetime even further. Exploration of energy transfer^1^,^37^ among different COPV*n* derivatives co-doped in the same film, or the use of longer COPV*n* with *n*>6 that emit at longer wavelengths will offer exciting future fields of investigation towards wavelength tunability. Truly continuous tunable laser devices will become available by combining the chemical tuning with the recent technological developments such as the use of active films with continuously variable thickness (that is, wedged configuration)^38^. Finally, the outstanding laser performances of COPV*n* offer promise not only for DFB lasers but also for organic solid-state lasers with other types of laser resonators^1^,^2^,^36^, as well as for the exploitation of new organic laser concepts^1^,^39^.

## Methods

### Synthesis

COPV*n* were synthesized from commercially available materials as previously described^24^. Structures of the COPV*n* along with their purity were confirmed by ^1^H and ^13^C NMR spectroscopy and mass spectrometry.

### Thin film preparation and DFB resonator fabrication

A thin film of COPV*n*-doped PS was prepared by spin-coating on a substrate. The percentage of PS in the solvent (toluene) was adjusted to control the film thickness between 0.6 and 0.7 μm (determined from the fringe pattern of the absorption spectrum). A commercially available FS substrate was used for absorption, PL and ASE measurements. For DFB laser fabrication, the film was deposited on a substrate of a different nature as required, with surface relief gratings previously recorded using HL or thermal-NIL. Grating dimensions were 2.5 × 2.5 cm and 2 × 2 mm for HL and thermal-NIL gratings, respectively. The grating used for the COPV1 laser was fabricated over a dichromated gelatine (DCG) layer and transferred to glass using reactive ion beam etching^40^. Gratings for COPV3–6 DFBs were prepared by thermal-NIL over a thermoplastic resist and transferred, by means of reactive ion etching^32^, to either FS substrates such as the ones used for ASE characterization, or alternatively to SiO_2_ layers over silicon. Both SiO_2_ and FS are transparent and have similar refractive index, so DFB performance is independent on the selection of one or another, so far as the laser characterization (excitation and collection) is performed from the side of the sample facing the active film (as was the case here). Gratings for COPV2 DFBs were prepared using HL over DCG or over dichromated poly(vinyl alcohol) (DCPVA) photoresist layers as recently reported^41^,^42^, which had been previously spin-coated over FS substrates.

### Optical characterization

For details of basic optical experiments (absorption and PL) and film PLQY measurements, see Supplementary Note 2. DFB and ASE characterizations were performed under optical excitation with a pulsed Nd:YAG (YAG (yttrium aluminium garnet)) laser (10 ns pulse width, 10 Hz repetition rate). For ASE, the pump beam (a stripe of dimensions 3.5 × 0.5 mm) was incident perpendicularly over the sample surface, and the emitted light was collected from the film edge with a fibre spectrometer (resolution 1.3 nm). For DFB characterization, the pump beam over the sample (elliptical with a minor axis of 1.1 mm and area of 1.0 mm^2^) was incident at ∼20° with respect to the normal to the film plane. This small deviation from normal incidence was chosen to facilitate light collection, by means of a 0.13 nm resolution fibre spectrometer, in a direction perpendicular to the sample surface. The pump size value was chosen as to be sufficiently large to ensure that the obtained laser threshold expressed in power or energy density units is a useful parameter for the sake of comparison^35^. The term pump intensity used throughout the manuscript refers to incident pump intensity. For both ASE and DFB studies, *λ*_pump_ was 355 nm for COPV1 and COPV2, 532 nm for COPV5 and COPV6 and 436 nm (provided by a Raman cell pumped with the 532 nm line of the Nd:YAG laser) for COPV3 and COPV4. For each of the concentrations and the compounds, we performed measurements on various nominally identical samples, aiming to ensure reproducibility of the ASE and DFB parameters.

## 

## Additional information

**How to cite this article:** Morales-Vidal, M. *et al*. Carbon-bridged oligo(*p*-phenylenevinylene)s for photostable and broadly tunable, solution-processable thin film organic lasers. *Nat. Commun*. 6:8458 doi: 10.1038/ncomms9458 (2015).

## Supplementary Material

Supplementary Information Supplementary Figures 1-3 and Supplementary Notes 1-2

## Figures and Tables

**Table 1:** Optical and amplified spontaneous emission (ASE) parameters of COPV*n*-doped polystyrene (PS) films deposited over fused silica.

*n*	COPV*n*- wt% in PS*	*h*† (μm)	PLQY (%)	*λ*_pump_‡ (nm)	*α*[*λ*_pump_]§ (cm^−1^)	*λ*_ASE_‖ (nm)	**I_th-ASE_¶ (kW cm^−2^)	*τ*_1/2_^ASE^# soft pump(pump pulses)	*τ*_1/2_^ASE^** extreme pump(pump pulses)	FWHM_ASE_†† (nm)
1	3.0	0.61	100	355	1.13 × 10^3^	384.5	250	1.2 × 10^2^	—	7
	5.0	0.72	100	355	1.88 × 10^3^	385.2	90	1.8 × 10^2^	—	6
2	3.0	0.61	100	355	6.0 × 10^2^	462.7	33	1.7 × 10^4^	1.2 × 10^3^	9
	5.0	0.68	100	355	9.3 × 10^2^	464.0	18	1.6 × 10^4^	—	6
3	2.0	0.65	100	436	1.91 × 10^3^	514.8	4.0	8.4 × 10^4^	6.0 × 10^3^	8
4	1.7	0.54	100	436	0.90 × 10^3^	548.2	8	2.2 × 10^5^	1.7 × 10^4^	9
	5.0	0.60	100	436	2.4 × 10^3^	549.6	2.7	1.4 × 10^5^	1.0 × 10^4^	8
	15.0	0.53	85	436	7.9 × 10^3^	552.2	1.5	8.0 × 10^4^	5.6 × 10^3^	7
5	0.5	0.63	100	532	50	571.1	90	3.0 × 10^5^	1.8 × 10^4^	8
	1.0	0.62	100	532	1.0 × 10^2^	571.4	55	3.7 × 10^5^	2.2 × 10^4^	8
6	0.5	0.62	100	532	3.9 × 10^2^	582.4	17	7.7 × 10^5^	5.1 × 10^4^	8
	1.0	0.64	94	532	6.1 × 10^2^	582.7	10	7.5 × 10^5^	4.7 × 10^4^	9
	2.0	0.69	99	532	1.6 × 10^3^	583.4	3.0	9.6 × 10^5^	5.0 × 10^4^	8
	4.0	0.77	94	532	2.89 × 10^3^	583.7	1.7	3.6 × 10^5^	2.4 × 10^4^	8
	8.0	0.52	78	532	6.5 × 10^3^	584.4	2.5	1.2 × 10^5^	1.3 × 10^4^	7
	20.1	0.49	40	532	1.59 × 10^4^	585.0	2.0	3.5 × 10^4^	2.5 × 10^3^	6

COPV, carbon-bridged oligo(*p*-phenylenevinylene); FWHM, full width at half maximum; PLQY, photoluminescence quantum yield (errors for COPV6 films in Fig. 2e).

^*^Error∼0.1%.

^†^Film thickness (error∼5%).

^‡^Pump wavelength.

^§^Absorption coefficient at the pump wavelength (errors in Fig. 2d).

^‖^ASE wavelength (error is ±0.5 nm).

^¶^ASE threshold (errors in Fig. 2d, estimated statistically as the s.d. from measurements on several nominally identical samples).

^#^ASE photostability half-life under I_pump_∼(2 × I_th-ASE_) at 10 Hz pump (error∼10%, estimated same as above).

^**^ASE photostability half-life under I_pump_=2.5 × 10^3^ kW cm^−2^ at 10 Hz pump (error∼10%).

^††^ASE linewidth (error is ±1 nm).

**Table 2:** Parameters of one-dimensional, second-order distributed feedback (DFB) lasers based on COPV*n*-doped polystyrene (PS) films.

COPV*n* device*	COPV*n*-wt% in PS†	*h*‡ (μm)	Resonator material	*d*§ (nm)	*Λ*‖ (nm)	*λ*_ASE_¶ (nm)	*λ*_DFB_ # (nm)	I_th-DFB_** (kW cm^−2^)	*τ*_1/2_^DFB^†† (pump pulses)
6^A^	0.5	0.62	SiO_2_	95	368	582.4	573.0	11	8.0 × 10^5^
6^B^	2.0	0.47	SiO_2_	30	380	583.4	579.6	2.1	1.0 × 10^6^
6^C^	8.0	0.49	SiO_2_	75	380	584.4	586.7	0.7	1.0 × 10^5^
6^D^	8.0	0.49	FS	60	380	584.4	586.6	0.7	1.1 × 10^5^
6^E^	20.1	0.57	FS	60	380	585.0	591.4	0.8	1.1 × 10^4^
5	1.0	0.63	SiO_2_	95	368	571.1	573.1	20	4.0 × 10^5^
4	5.0	0.60	SiO_2_	73	353	549.2	556.6	1.0	3.9 × 10^5^
3	2.0	0.65	SiO_2_	78	321	514.8	516.4	1.0	3.0 × 10^5^
2^A^	5.0	0.65	DCG	50	308	464.0	490.9	90	4.0 × 10^3^
2^B^	5.0	0.38	DCPVA	40	296	464.0	464.7	550	8.0 × 10^2^
1	3.0	0.61	Glass	70	270	384.5	407.8	>8,000	—

COPV, carbon-bridged oligo(p-phenylenevinylene); DCG, dichromated gelatine photoresist layer over FS; DCPVA, dichromated poly(vinyl alcohol) photoresist layer over FS; FS, fused silica; SiO_2_, SiO_2_ layer over silicon.

^*^Number on the label refers to *n*.

^†^Error∼0.1%.

^‡^Film thickness (error∼5%).

^§^Grating depth.

^‖^Grating period.

^¶^ASE wavelength (error is ±0.5 nm).

^#^DFB wavelength (error is ±0.1 nm).

^**^DFB threshold. Error∼10%, estimated statistically as the s.d. from measurements on nominally identical samples.

^††^DFB photostability half-life under I_pump_∼(2 × *I*_th-DFB_) at 10 Hz. Error∼10%, estimated statistically as the s.d. from measurements on nominally identical samples.

## References

[b1] Chénais S. & Forget S. Recent advances in solid-state organic lasers. Polym. Int. 61, 390–406 (2012).

[b2] Grivas C. & Pollnau M. Organic solid-state integrated amplifiers and lasers. Laser Photon. Rev. 6, 419–462 (2012).

[b3] Camposeo A., Del Carro P., Persano L. & Pisignano D. Electrically tunable organic distributed feedback lasers embedding nonlinear optical molecules. Adv. Mater. 24, OP221–OP225 (2012). 22807224 10.1002/adma.201201453

[b4] Vannahme C., Klinkhammer S., Lemmer U. & Mappes T. Plastic lab-on-a-chip for fluorescence excitation with integrated organic semiconductor lasers. Opt. Express 19, 8179–8186 (2011). 21643068 10.1364/OE.19.008179

[b5] Clark J. & Lanzani G. Organic photonics for communications. Nat. Photon. 4, 438–446 (2010).

[b6] Wang Y. . LED pumped polymer laser sensor for explosives. Laser Photon. Rev. 7, L71–L76 (2013). 25821526 10.1002/lpor.201300072 PMC4374702

[b7] Heydari E. . Label-free biosensor based on an all-polymer DFB laser. Adv. Opt. Mater. 2, 137–141 (2014).

[b8] Guo L. J. Nanoimprint lithography: methods and material requirements. Adv. Mater. 19, 495–513 (2007).

[b9] Karnutsch C. . Low threshold blue conjugated polymer lasers with first- and second-order distributed feedback. Appl. Phys. Lett. 89, 201108 (2006).

[b10] Karnutsch C. . Improved organic semiconductor lasers based on a mixed-order distributed feedback resonator design. Appl. Phys. Lett. 90, 131104 (2007).

[b11] Yap B. K. . Simultaneous optimization of charge-carrier mobility and optical gain in semiconducting polymer films. Nat. Mater. 7, 376–380 (2008). 18408724 10.1038/nmat2165

[b12] Xia R., Lai W.-L., Levermore P. A., Huang W. & Bradley D. D. C. Low-threshold distributed-feedback lasers based on pyrene-cored starburst molecules with 1,3,6,8-attached oligo(9,9-dialkylfluorene) arms. Adv. Funct. Mater. 19, 2844–2850 (2009).

[b13] Yang Y., Turnbull G. A. & Samuel I. D. Hybrid optoelectronics: a polymer laser pumped by a nitride light-emitting diode. Appl. Phys. Lett. 92, 163306 (2008).

[b14] Herrnsdorf J. . Micro-LED pumped polymer laser: a discussion of future pump sources for organic lasers. Laser Photon. Rev. 7, 1065–1078 (2013).

[b15] Tsiminis G. . Nanoimprinted organic semiconductor laser pumped by a light-emitting diode. Adv. Mater. 25, 2826–2830 (2013). 23580437 10.1002/adma.201205096

[b16] Yang Y. . Highly photostable dye doped solid-state distributed-feedback (DFB) channeled waveguide lasers by a pen-drawing technique. Opt. Express 18, 22080–22089 (2010). 20941109 10.1364/OE.18.022080

[b17] Navarro-Fuster V. . Highly photostable organic distributed feedback laser emitting at 573 nm. Appl. Phys. Lett. 97, 171104 (2010).

[b18] Ramírez M. G. . 1,7-Bay-substituted perylenediimide derivative with outstanding laser performance. Adv. Opt. Mater. 1, 933–938 (2013).

[b19] Ramírez M. G. . Improved performance of perylenediimide-based lasers. J. Mater. Chem. C 1, 1182–1191 (2013).

[b20] Ramírez M. G. . Efficient organic distributed feedback lasers with imprinted active films. Opt. Express 19, 22443–22454 (2011). 22109121 10.1364/OE.19.022443

[b21] Laquai E. . Photophysical properties of a series of poly(ladder-type phenylene)s. Adv. Funct. Mater. 17, 3231–3240 (2007).

[b22] Choi E. Y. . Photophysical, amplified spontaneous emission and charge transport properties of oligofluorene derivatives in thin films. Phys. Chem. Chem. Phys. 16, 16941–16956 (2014). 25005146 10.1039/c4cp01134a

[b23] Ishow E. . Multicolor emission of small molecule-based amorphous thin films and nanoparticles with a single excitation wavelength. Chem. Mater. 20, 6597–6599 (2008).

[b24] Zhu X., Tsuji H., López-Navarrete J. T., Casado J. & Nakamura E. Carbon-bridged oligo(phenylenevinylene)s: stable π-systems with high responsiveness to doping and excitation. J. Am. Chem. Soc. 134, 19254–19259 (2012). 23106224 10.1021/ja309318s

[b25] Zhu X., Mitsui C., Tsuji H. & Nakamura E. Modular synthesis of 1*H*-indenes, dihydro-*s*-indacene, and diindenoindacene–a carbon-bridged *p*-phenylenevinylene congener. J. Am. Chem. Soc. 131, 13596–13597 (2009). 19728709 10.1021/ja905626b

[b26] Mroz M. M. . Amplified spontaneous emission in conjugated polyrotaxanes under quasi-cw pumping. Adv. Mater. 25, 4347–4351 (2013). 23813773 10.1002/adma.201301703

[b27] Sugiyasu K. . A self-threading polythiophene: defect-free insulated molecular wires endowed with long effective conjugation lengths. J. Am. Chem. Soc. 132, 14754–14756 (2010). 20879791 10.1021/ja107444m

[b28] Díaz-García M. A. . Concentration dependence of amplified spontaneous emission in two oligo-p-phenylenevinylene derivatives. J. Appl. Phys. 97, 063522_1–063522_6 (2005).

[b29] Mayorga Burrezo P. . Planarization, fusion, and strain of carbon-bridged phenylenevinylene oligomers enhance π-electron and charge conjugation: a dissectional vibrational Raman study. J. Am. Chem. Soc. 137, 3834–3843 (2015). 25730496 10.1021/ja5125463

[b30] Calzado E. M. . Amplified spontaneous emission in polymer films doped with a perylenediimide derivative. Appl. Opt. 46, 3836–3842 (2007). 17538681 10.1364/ao.46.003836

[b31] Xia R., Heliotis G., Hou Y. & Bradley D. D. C. Fluorene-based conjugated polymer optical gain media. Org. Electron. 4, 165–177 (2003).

[b32] Navarro-Fuster V. . Film thickness and grating depth variation in organic second-order distributed feedback lasers. J. Appl. Phys. 112, 043104 (2012).

[b33] Langhals H. Control of the interactions in multichromophores: novel concepts. Perylene bis-imides as components for larger functional units. Helv. Chim. Acta 88, 1309–1343 (2005).

[b34] Martins E. R. . Low-threshold nanoimprinted lasers using substructured gratings for control of distributed feedback. Adv. Opt. Mater. 1, 563–566 (2013).

[b35] Calzado E. M. . Influence of the excitation area on the thresholds of organic second-order distributed feedback lasers. Appl. Phys. Lett. 101, 223303 (2012).

[b36] Sastre R. . Dye-doped polyhedral oligomeric silsesquioxane (poss)-modified polymeric matrices for highly efficient and photostable solid-state lasers. Adv. Funct. Mater. 19, 3307–3316 (2009).

[b37] Casado J. . Amplified spontaneous emission in pentathienoacene dioxides by direct optical pump and by energy transfer: correlation with photophysical parameters. Adv. Opt. Mater. 1, 588–599 (2013).

[b38] Klinkhammer S. . Continuously tunable solution-processed organic semiconductor DFB lasers pumped by laser diode. Opt. Express 20, 6357–6364 (2012). 22418517 10.1364/OE.20.006357

[b39] Noginov M. A. . Demonstration of a spaser-based nanolaser. Nature 460, 1110–1112 (2009). 19684572 10.1038/nature08318

[b40] Calzado E. M. . Blue surface-emitting distributed feedback lasers based on TPD-doped films. Appl. Opt. 49, 463–470 (2010). 20090812 10.1364/AO.49.000463

[b41] Ramírez M. G. . Perylenediimide-based distributed feedback lasers with holographic relief gratings on dichromated gelatine. J. Appl. Phys. 114, 033107 (2013).

[b42] Ramírez M. G., Villalvilla J. M., Quintana J. A., Boj P. G. & Díaz-García M. A. Distributed feedback lasers based on dichromated poly(vinyl alcohol) reusable surface-relief gratings. Opt. Mater. Express 4, 733–738 (2014).

