# Supplementary material for: Carbon-bridged oligo(p-phenylenevinylene)s for photostable and broadly tunable, solution-processable thin film organic lasers
Source: Nat Commun. 2015 Sep 29;6:8458. doi: 10.1038/ncomms9458 (PMC4598723; doi:10.1038/ncomms9458)
Supplement: Supplementary Information — Supplementary Figures 1-3 and Supplementary Notes 1-2 [file ncomms9458-s1.pdf]

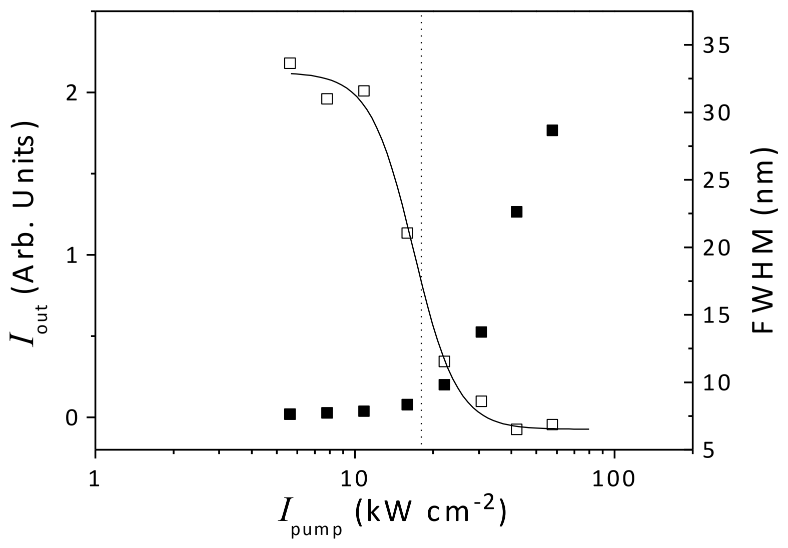

Supplementary Figure 1. Amplified spontaneous emission (ASE) plots used for threshold determination. The numerical value of the ASE threshold for a given film was determined from the plot of its emission linewidth (defined as the full with at half of maximum intensity, FWHM) versus pump intensity (I_{pump}), as the I_{pump} value at which FWHM decays to half of its maximum value. Such a plot for a 5.0 wt% COPV2-doped PS film is represented by open squares in the right axis of the figure. The full line is a guide to the eye. ASE threshold determination from the plot of the output intensity (I_{out}) at the wavelength at which ASE appears versus I_{pump} (such a plot for the 5.0-wt% COPV2-doped PS film is represented by full squares in the left axis), as the I_{pump} value at which a drastic slope change occurs (method used for DFB threshold determination) would involve larger errors, since the change in slope is gradual and hence not well-defined.

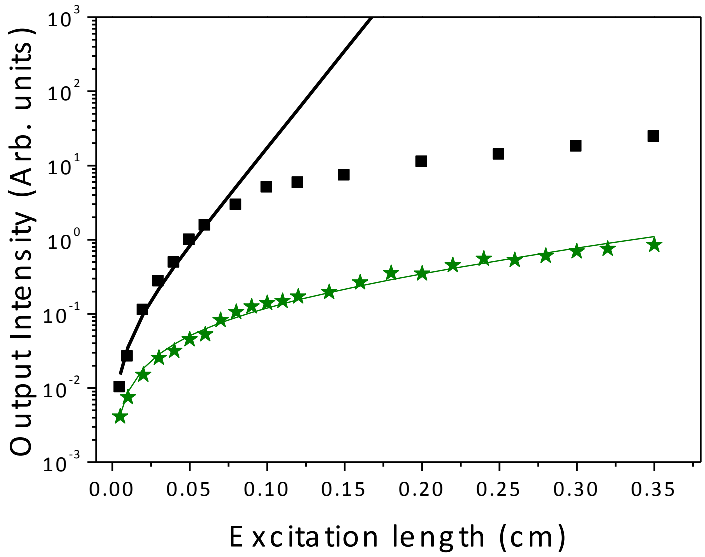

Supplementary Figure 2. Amplified spontaneous emission (ASE) Variable Length Stripe study for net gain coefficients determination. Emission intensity at the wavelength at which ASE appears (λ = 584 nm) versus the length of the excitation stripe for an 8 wt% COPV6-doped PS film at pump intensities of 43.3 and 11.5 kW cm^{-2} (squares and starts respectively). The solid lines are fits to the data using Supplementary Equation 1 (see Supplementary note 1), from which net gain coefficients, *g*, of 60 and 6.3 cm^{-1}, respectively, were obtained.

In the curve obtained at the highest pump intensity (43.3 kW cm^{-2}) gain saturation is present, so only points obtained at short excitation lengths were used for the fit.

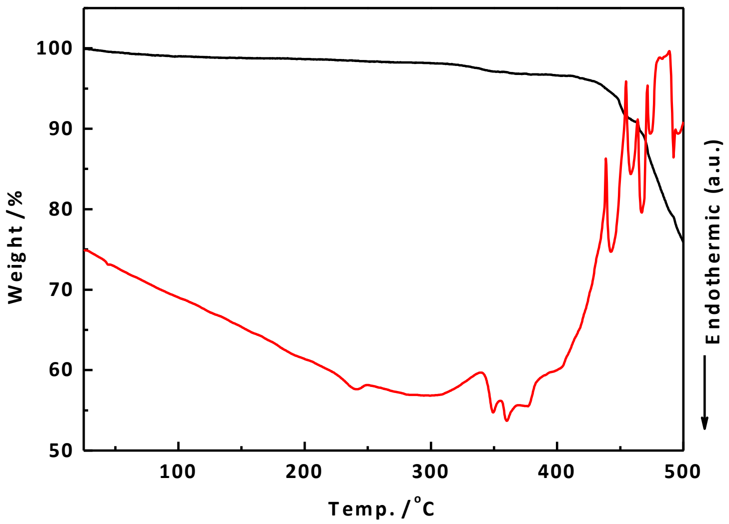

Supplementary Figure 3. Thermogravimetric (TG) and differential thermal analysis (DTA) traces for COPV6. Experiments were carried out with a Rigaku TG8120 apparatus. Temperature was raised at 10 K min^{-1} under a N_2 flux of 100 mL min^{-1}. Temperature difference from a reference sample (Al_2O_3) and relative weights were measured as a function of temperature as shown. Decomposition temperature (5% weight loss) was found at 439 °C.

Supplementary Note 1: Amplified spontaneous emission (ASE) Variable Stripe Length study for net gain coefficients determination.

When ASE is the mechanism responsible for the observation of spectral gain narrowing and of a sudden increase of the output intensity at a given pump intensity (the ASE threshold), the output intensity at the end of the excitation stripe should follow the expression:

$$I(\lambda) = \frac{A(\lambda)I_{\mathrm{pump}}}{g(\lambda)}\left(e^{g(\lambda)l} - 1\right) \qquad \text{(Supplementary Equation 1)}$$

where A is a is a wavelength (λ) dependent constant, related to the cross section for spontaneous emission; I_{pump} is the pump intensity; g is the net gain coefficient, which depends on λ; and l is the length of the pump stripe. Note that this expression does not have into account saturation effects appearing at high pump intensities.

The net gain coefficient for a given pump intensity can be determined by fitting the output intensity at the peak of the emission spectrum as a function of the pump stripe length. An example of such a study is shown in Supplementary Figure 2 for an 8 wt% COPV6-doped PS film at two pump intensities, 43.3 and 11.5 kW cm^{-2}. Data have been fitted with Supplementary Equation 1, using AI_{p} values of 2.60 and 0.86 (in arbitrary units), and net gain coefficients of 60 and 6.3 cm^{-1}, respectively.

Supplementary Note 2: Absorption, PL and PLQY

Absorption measurements were carried out in a Jasco V-650 spectrophotometer. The absorption coefficient for a film of thickness (h) at a given wavelength (α_λ) was calculated according to $\alpha_\lambda = 2.3\ A_\lambda / h$, where A_λ is the absorbance. Standard PL spectra were obtained in a Jasco FP-6500 fluorimeter by exciting at a 60º angle with respect to the normal to the film. PL emission was collected in reflection at a 30º angle, in order to avoid the pump beam. The excitation wavelength was that at which the PL intensity was maximum in each case (336, 379, 438, 436, 478, 490 nm for films doped with COPV1, 2, 3, 4, 5, and 6, respectively). PLQY of COPV films were measured using a Jasco ISF-513 integrating sphere mounted into the Jasco FP-6500 fluorimeter. The excitation wavelengths were the same as those used in PL measurements.
